# Success rates of single-thread and double-thread orthodontic miniscrews in the maxillary arch

**DOI:** 10.1186/s12903-024-03866-x

**Published:** 2024-02-05

**Authors:** Mohsen Merati, Hassanali Ghaffari, Fatemeh Javid, Farzaneh Ahrari

**Affiliations:** 1 Department of Orthodontics, School of Dentistry, Shahed University, Tehran, Iran; 2 School of Dentistry, Shahed University of Medical Sciences, Tehran, Iran; 3 Dental Research Center, School of Dentistry, Mashhad University of Medical Sciences, Vakilabad Blvd, Mashhad, Iran

**Keywords:** Dental implant, Miniscrew, Orthodontic anchorage, Orthodontic appliances, Orthodontic treatment, Success rate, Survival rate

## Abstract

**Aim:**

There is limited research on the clinical performance of double-thread orthodontic miniscrews. This study aimed to compare the stability of double-thread and single-thread orthodontic miniscrews and identify the potential associations between patient-related and location-related factors with miniscrew stability.

**Methods:**

This retrospective cohort study involved 90 orthodontic miniscrews (45 single-thread, 45 double-thread) with identical dimensions (8 mm length, 1.6 mm diameter). The screws were inserted in various locations within the upper jaw of 83 patients (54 females, 29 males; mean age = 15.1 ± 2.4 years). Failure was defined as excessive mobility or loss of miniscrew after placement. The data recorded were patient age, gender, insertion site, side of insertion (buccal or lingual), duration of force application, and failure occurrence.

**Results:**

The overall success rate within the sample was 92.2%. Double-thread miniscrews exhibited a significantly higher success rate than single-thread miniscrews (*P* = 0.049), with 97.8% and 86.7% success rates, respectively. Gender, age, insertion location, and side of insertion did not show significant associations with failure (*P* > 0.05). Log-rank analysis revealed a significant difference between the two groups (*P* = 0.046), indicating a higher probability of survival for the double-thread design.

**Conclusions:**

The overall success rate of orthodontic miniscrews was high in the present sample. Double-thread miniscrews placed in various locations within the maxillary arch demonstrated superior stability and survival rates compared to their single-thread counterparts. Therefore, double-thread miniscrews may be preferred when bone quality is inadequate, such as in young patients.

## Introduction

The introduction of intra-bony implants by Branemark marked significant progress in various dental fields, including orthodontics. A temporary anchorage device (TAD) is a type of mini-implant inserted into the bone to overcome the longstanding challenge of anchorage control during orthodontic therapy and is removed after treatment completion. TADs are replacing conventional methods of anchorage control such as extra-oral appliances or trans palatal arches, helping to provide satisfactory outcomes in complex cases without compromising facial aesthetics or relying on patient cooperation [1–3].

Among various types of TADs, miniscrews are the most widely used in clinical practice due to their small size, ease of insertion and removal, and cost-effectiveness compared to other systems [4, 5]. Miniscrews enable efficient and versatile tooth movements such as intrusion or retraction of anterior and posterior teeth, correction of canted occlusal plane, unilateral space closure, and alignment of dental midlines [6–9].

Unlike osseointegrated implants, miniscrews do not require osseointegration and are typically loaded immediately after insertion. Therefore, achieving primary stability is crucial for successfully using miniscrews as a skeletal anchorage system [4]. Primary stability mainly depends on mechanical interlocking between the screw threads and the surrounding bone tissue [10]. However, miniscrews can sometimes become mobile and fail shortly after insertion. The success rate of miniscrews ranges from 60 to 93% [10, 11], which is lower than osseointegrated implants (96–99%) [3]. Various factors influence the success rate of miniscrews, including bone quality and quantity at the insertion site, smoking, screw diameter and length, thread design, surgical technique, jaw, and oral hygiene status [3, 6, 7, 12].

Maximizing the contact area between the screw and alveolar bone is an effective strategy to improve the primary stability of TADs, particularly in cases with inadequate bone quality or quantity. This goal may be achieved by increasing the diameter and length of the mini-implant [13, 14]. However, placing very long or thick screws is often impossible due to anatomical limitations and the risk of root contact. An alternative strategy is modifying the thread design to enhance mechanical retention and contact area with the alveolar bone. Double-thread miniscrews were developed to provide more excellent mechanical interlocking than traditional single-thread screws. The presence of micro threads in the upper part of the screw increases the contact area with the cortical bone, thereby improving stress distribution and primary stability of the mini-implant [5, 12, 14].

Previous studies have investigated the effect of various variables such as length, diameter, and thread features (shape, pitch, depth) on the primary stability of mini-implants in laboratory conditions [3, 4, 15–21] or animal studies [22–25]. However, it is essential to note that the mechanical properties of artificial bone and the bone quality of animals differ from humans [10, 14]. There is limited research on the clinical performance of double-thread compared to single-thread miniscrews. This study aimed to assess the clinical success rate and survival duration of double-thread and single-thread miniscrews and identify potential associations between patient-related (age, gender) and location-related (placement area and side) parameters with miniscrew stability.

## Materials and methods

This retrospective cohort study included the records of patients who received miniscrews from March 2016 to August 2019. The inclusion criteria included inserting single-thread or double-thread miniscrews with identical specifications (8 mm length, 1.6 mm diameter) in various locations within the upper jaw. The treatment procedures were conducted in a private orthodontic clinic. Patients with maternal anomalies or systemic diseases, as well as smokers, or those using alcohol or medications affecting tooth movement, were excluded. The study protocol received approval from the ethics committee of Shahed University. Informed consent was obtained from all patients or their parents / legal guardians (if their age was less than 16 years old), adhering to the principles of the Helsinki Declaration.

**Fig. 1:**
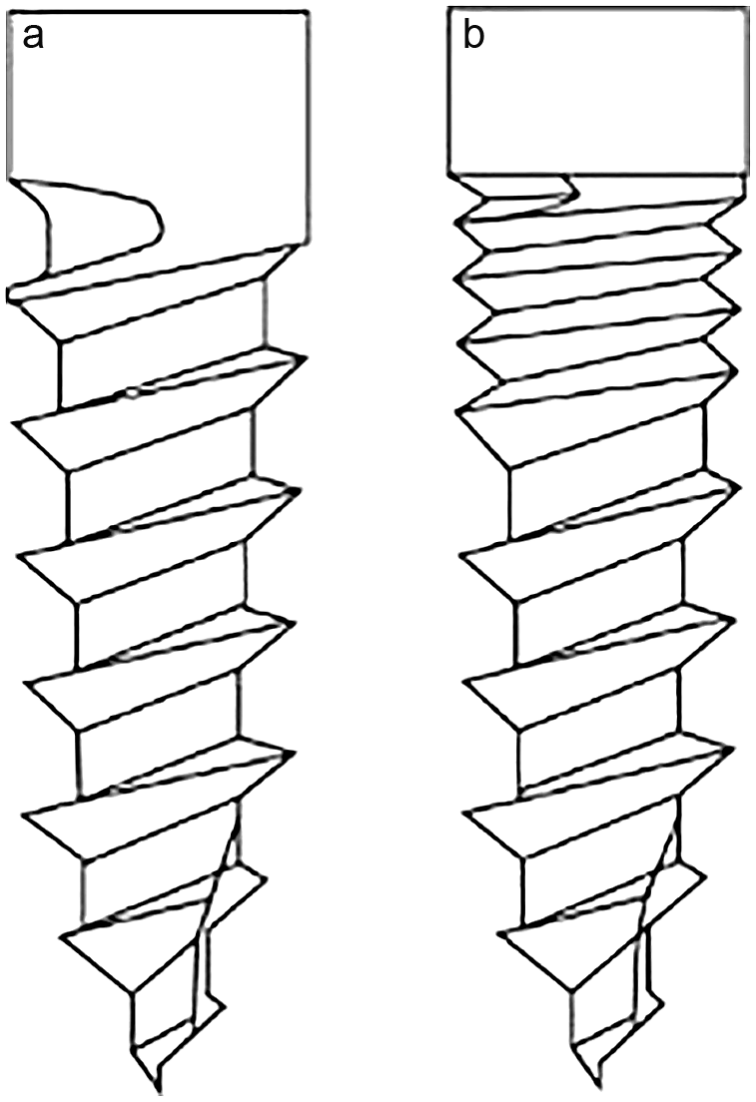
A schematic illustration of single-thread (left) and double-thread (right) miniscrews

The study sample was divided into two groups based on the design of the inserted miniscrews. The first group comprised 45 single-thread miniscrews (Jeil Medical Corporation, Seoul, Korea), while the second group included 45 double-thread miniscrews (KJ Meditech, Gwangju, Korea). All screws were fabricated from titanium alloy (Ti-6Al-4 V), featured a tapered shape, and had no surface treatment. These miniscrews were placed in 83 patients (54 females, 29 males) with a mean age of 15.1 ± 2.4 years, ranging from 12 to 23 years. Figure 1 illustrates the design of the miniscrews used in this study. The double-thread design featured micro threads in the upper portion of the miniscrew, exhibiting approximately half the pitch of the lower threads.

Miniscrews were inserted after levelling and aligning teeth in various upper jaw regions. A skilled orthodontist performed the surgical procedures under local anesthesia without predrilling or incision. A force of 150 g was applied immediately after surgery through NiTi closed coil springs. Patients were instructed to maintain proper oral hygiene and avoid striking the screws. Follow-up appointments were scheduled every four weeks, and failure was defined as the presence of excessive mobility or loss of a screw after insertion.

Data extracted from patients records were categorized into patient-related factors (age and gender), location-related factors (insertion site and side), and screw design factors (single-thread or double-thread). The duration of force application and occurrence of failure were recorded for each screw.

### Statistical analysis

The chi-square test was applied to compare the success rate between the two screw designs (single-thread versus double-thread). The association between the miniscrew success rate and various variables, including gender, age, placement area, and insertion side (buccal or lingual), was assessed using the chi-square or Fisher’s exact test, when appropriate. The survival rate of miniscrews was compared between the two screw designs using the log-rank test. The statistical analysis was performed by SPSS software, version 16.0 (SPSS, Chicago, IL), with a significance level set at *P* < 0.05.

## Results

In this study, 90 miniscrews were inserted into various areas of the maxilla. Seven failures were observed in the sample, resulting in an overall success rate of 92.2% (83 out of 90 miniscrews). The earliest failure occurred after 3 days, while the latest was observed on day 45 following screw placement and force application. The minimum and maximum periods of force application to miniscrews ranged from 3 to 8 months.

Table 1 compares the success rate between single-thread and double-thread miniscrews within the sample. Failures were noted in 1 of the double-thread and 6 of the single-thread miniscrews. Statistical analysis using the chi-square test demonstrated a significant difference between the two designs (*P* = 0.049; Table 1), with the double-thread group exhibiting a significantly higher success rate (97.8%) than the single-thread (86.7%) design.

**Table 1:** Success rates of the single-thread and double-thread designs of miniscrews

	Success (N)	Failure (N)	Success rate (%)	*P*-value
Single-thread	39	6	86.7	0.049
Double-thread	44	1	97.8	
Overall	83	7	92.2	

Table 2 outlines the association between the overall success rate of the miniscrews and some patient-related and location-related factors. There were no statistically significant differences in the success rate of the miniscrews between male and female patients (*P* = 0.242, Table 2) or between patients older or younger than 16 years of age (*P* = 0.723, Table 2). Additionally, the area of placement and the side of placement (buccal or lingual) did not significantly influence the success rate of miniscrews (*P* = 0.506 and *P* = 0.522, respectively; Table 2).

**Table 2:** The association between the success rate of the miniscrews in the upper jaw and some patient-related and location-related variables

		Success (N)	Failure (N)	Success rate (%)	*P*-value
Gender	Female	54	3	94.7	0.242
	Male	29	4	87.9	
Age	< 16 years	53	4	93.0	0.723
	> 16 years	30	3	90.9	
Area	2–3	5	0	100	0.506
	5–6	51	3	94.4	
	6–7	27	4	87.1	
Side	Buccal of maxilla	46	3	93.9	0.522
	Palatal of maxilla	37	4	90.2	

Table 3 presents the survival periods of the two screw designs at a 95% confidence interval. The estimated survival time was 194 days for the single-thread group and 239 days for the double-thread group. The log-rank analysis demonstrated a significant difference in the survival rate between the two groups (*P* = 0.046), indicating a higher probability of survival for the double-thread design.

**Table 3:** The survival distribution of the single-thread and double-thread miniscrews

Group	Estimate	Standard error	95% Confidence Interval	
			Lower bound	Upper bound
Conventional	194.52	10.147	174.630	214.407
Double-thread	239.71	5.230	229.461	249.962
Overall	227.66	6.344	215.226	240.093

## Discussion

The present study evaluated the clinical stability and survival of double-thread versus single-thread miniscrews while examining the impact of specific patient-related factors (gender and age) and location-related factors (area and side of insertion) on miniscrew success rates. The inclusion criteria were carefully structured to ensure that screw-related parameters, except the thread design, were consistent in both groups (8 mm length, 1.6 mm diameter). The evaluation was limited to machined-surface screws placed in the maxilla to minimize the impact of variables like surface treatment, cortical bone thickness, the quantity of keratinized tissue, and jaw vascularization on the miniscrew outcomes. Exclusion criteria effectively controlled for confounding variables such as systemic diseases and heavy smoking through precise case selection.

The orthodontic force of 150 g was immediately applied post-insertion using NiTi open coil springs. While there is some debate regarding the influence of immediate versus delayed loading on the survival rate of miniscrews [6], several studies suggest that immediate loading may enhance cellular turnover, resulting in comparable or improved results compared to implants with forces applied later [6, 26–29]. Manni et al. [6] recommended the application of immediate forces not exceeding 150–250 g to the screw.

In the present study, the overall success rate of the screws inserted in the maxillary arch was 92.2%, as defined by the absence of screw mobility during orthodontic force application. All failures occurred within 45 days of miniscrew insertion. Various factors have been proposed to contribute to miniscrew failure, including excessive loading, unscrewing due to interacting forces, inflammation around the screw, and application of torquing forces [6]. While the literature reports a wide range of success rates, most studies indicate success rates exceeding 80% for temporary anchorage devices [6, 10–12, 30–32]. Variability in success rates can be attributed to differences in miniscrew designs and criteria for defining treatment success, as well as variations in host and location factors among the studies.

Failure was observed in 6 screws from the single-thread group and 1 screw from the double-thread group. Statistical analysis revealed a significantly higher success rate for double-thread screws (97.8%) compared to single-thread screws (86.7%). Additionally, the probability of survival was significantly greater for double-thread screws (239 days) than for single-thread screws (194 days), indicating enhanced stability for the double-thread design in clinical conditions. These results suggest that using double-thread miniscrews in the maxilla significantly improves the success rate and ensures better implant survival until achieving treatment objectives, compared to conventional screws. The improved success rate of the double-thread design may be attributed to increased contact with the cortical bone provided by the upper micro threads, which enhances stress distribution and primary stability of the mini-implant [5, 12, 14].

The use of double-thread screws may be particularly advantageous in cases with limited quantity or quality of alveolar bone. Some examples are young patients with incomplete bone maturation or when anatomical constraints require the use of a small-diameter or short mini-implant [12, 14]. However, placing double-thread screws is associated with high insertion and removal torque, which can lead to overheating during insertion, excessive stress on the surrounding bone, and screw fracture in cases with thick cortical bone or high bone density [5, 14]. Therefore, it is recommended to limit the use of these screws to areas with lower bone density, especially in the maxilla. In contrast, the single-thread design may be more appropriate for the mandible, where the cortical bone is thicker and denser [5, 12].

The findings of this study align with previous studies that demonstrated improved mechanical properties of double-thread mini-implants [5, 14, 33]. Cha et al. [5] reported significantly higher maximum insertion torque with dual-thread than single-thread screws across all cortical bone thicknesses. Kim et al. [14] investigated various shapes of mini-implants, including cylindrical, taper, and dual-thread. They found that the dual-thread shape exhibited a gradual increase in insertion torque and a gentle decrease in removal torque compared to other designs. However, they argued that the dual-thread shape may need refinement to reduce insertion time and minimize stress on surrounding tissues [14].

In contrast to the findings of this study, Fukumoto et al. [34] exhibited comparable survival and bone-miniscrew contact (BMSC) rates in single- and dual-thread miniscrews placed on the palatal aspect of the maxillary tuberosity. Durrani et al. [20] indicated no significant difference in failure rates between dual-thread and single-thread TADs. Lee et al. [12] found comparable success rates of 82.1% and 84.4% for cylindrical and dual-thread miniscrews, respectively. They concluded that dual-thread miniscrews were not superior to cylindrical ones in terms of the long-term stability and clinical success rate [12].

In this study, the clinical success rate did not significantly differ between female (94.7%) and male (87.9%) subjects, which aligns with findings from most studies in the literature [11, 32, 34–36]. However, Manni et al. [6] reported a better success rate in males (88.1%) than in females (76.4%). They attributed this difference to the large sample size of their study and variations in cortical bone thickness and hormonal status between genders. The association between age and success rate was insignificant in this study; the success rate in patients younger than 16 years (93.0%) was comparable to those over 16 (90.9%). Some studies have also reported no significant difference in success rate between ages [11, 32, 36]. In contrast, several studies have found that adolescent patients have lower success rates with orthodontic miniscrews than older individuals [10, 12, 30, 37, 38]. This difference was attributed to lower bone density, thinner cortical bone, and higher bone turnover in growing subjects [10, 12]. The similar success rates across different ages observed in this study may be related to the use of double-thread screws in half of the patients, which provides more excellent mechanical stability than conventional screws, thus ensuring high clinical success even in young patients.

In this study, the screws were inserted in various segments of the upper arch: 5 screws between the lateral incisor and canine teeth, 54 miniscrews between the second premolars and first molars, and 31 screws between the first and second molars. The success rate of screws placed in different areas of the jaw did not show a significant difference. The success rate of screws located on the buccal side (46/49) was similar to those on the palatal side (37/41), and the association between the side of insertion and screw stability was not significant. Similar findings have also been reported in other studies [11, 32].

The limitations of this study were the relatively small sample size and the variability in force vector among the patients. Furthermore, observational retrospective studies are prone to selection bias, which could impact the generalizability of findings and potentially affect the differences in success rates between the two types of miniscrews. More extensive split-mouth studies are recommended to assess the primary and long-term stability of double-thread miniscrews compared to other screw types, helping to select the most suitable design for clinical practice.

## Conclusions

1. The overall success rate of the screws inserted in the maxillary arch was 92.2% (83/90 miniscrews). The double-thread screws exhibited a significantly higher success rate compared to the single-thread design (97.8% versus 86.7%).
2. Factors related to patients (age and gender) and the location of insertion (area and side) did not demonstrate any significant associations with the success rate of screws in the upper jaw.

## Data Availability

The datasets used and/or analyzed during the current study are available from the corresponding author upon reasonable request.
